# Anti-CX3CL1 (fractalkine) monoclonal antibody attenuates lung and skin fibrosis in sclerodermatous graft-versus-host disease mouse model

**DOI:** 10.1186/s13075-024-03307-8

**Published:** 2024-05-03

**Authors:** Takumi Hasegawa, Akira Utsunomiya, Takenao Chino, Hiroshi Kasamatsu, Tomomi Shimizu, Takashi Matsushita, Takashi Obara, Naoto Ishii, Hideaki Ogasawara, Wataru Ikeda, Toshio Imai, Noritaka Oyama, Minoru Hasegawa

**Affiliations:** 1 Department of Dermatology, Division of Medicine, Faculty of Medical Sciences, University of Fukui, Fukui, 910-1193 Japan; 2 Department of Dermatology, Faculty of Medicine, Institute of Medical, Pharmaceutical and Health Sciences, Kanazawa University, Kanazawa, 920-8641 Japan; 3 Eisai Co., Ltd, Tsukuba, 300-2635 Japan; 4 KAN Research Institute, Inc, Kobe, Hyogo 650-0047 Japan; 5 Present Address: IDDK Co., Ltd, Tokyo, 135-0047 Japan; 6 Present Address: Advanced Therapeutic Target Discovery, Department of Gastroenterology, Kobe University Graduate School of Medicine, Kobe, Hyogo 650-0047 Japan

**Keywords:** 3 ~ 10, CX3CL1, CXCR1, Fibrosis, Fractalkine, Graft-versus-host disease, Lung, Scleroderma, Skin, Systemic sclerosis

## Abstract

**Background:**

Systemic sclerosis (SSc) is an autoimmune disease characterized by vascular injury and inflammation, followed by excessive fibrosis of the skin and other internal organs, including the lungs. CX3CL1 (fractalkine), a chemokine expressed on endothelial cells, supports the migration of macrophages and T cells that express its specific receptor CX3CR1 into targeted tissues. We previously reported that anti-CX3CL1 monoclonal antibody (mAb) treatment significantly inhibited transforming growth factor (TGF)-β1-induced expression of type I collagen and fibronectin 1 in human dermal fibroblasts. Additionally, anti-mouse CX3CL1 mAb efficiently suppressed skin inflammation and fibrosis in bleomycin- and growth factor-induced SSc mouse models. However, further studies using different mouse models of the complex immunopathology of SSc are required before the initiation of a clinical trial of CX3CL1 inhibitors for human SSc.

**Methods:**

To assess the preclinical utility and functional mechanism of anti-CX3CL1 mAb therapy in skin and lung fibrosis, a sclerodermatous chronic graft-versus-host disease (Scl-cGVHD) mouse model was analyzed with immunohistochemical staining for characteristic infiltrating cells and RNA sequencing assays.

**Results:**

On day 42 after bone marrow transplantation, Scl-cGVHD mice showed increased serum CX3CL1 level. Intraperitoneal administration of anti-CX3CL1 mAb inhibited the development of fibrosis in the skin and lungs of Scl-cGVHD model, and did not result in any apparent adverse events. The therapeutic effects were correlated with the number of tissue-infiltrating inflammatory cells and α-smooth muscle actin (α-SMA)-positive myofibroblasts. RNA sequencing analysis of the fibrotic skin demonstrated that cGVHD-dependent induction of gene sets associated with macrophage-related inflammation and fibrosis was significantly downregulated by mAb treatment. In the process of fibrosis, mAb treatment reduced cGVHD-induced infiltration of macrophages and T cells in the skin and lungs, especially those expressing CX3CR1.

**Conclusions:**

Together with our previous findings in other SSc mouse models, the current results indicated that anti-CX3CL1 mAb therapy could be a rational therapeutic approach for fibrotic disorders, such as human SSc and Scl-cGVHD.

**Supplementary Information:**

The online version contains supplementary material available at 10.1186/s13075-024-03307-8.

## Background

Systemic sclerosis (SSc) is a collagen disease that affects the skin and various internal organs. Of note, lung involvement in SSc represents a principal cause of mortality. SSc pathogenesis is characterized by vasculopathy, inflammation, and subsequent fibrosis in the target tissues [1]. Histological analysis of early stage SSc skin showed perivascular infiltrates consisting of macrophages and T cells [2, 3]. Increasing evidence for a pathogenic role of macrophages in SSc has attracted attention regarding excessive fibrosis and relevant inflammatory processes owing to their production of various profibrotic molecules, including IL-6, transforming growth factor (TGF)-β, and osteopontin (Spp1) [4]. In SSc patients, the number of non-classical patrolling monocytes increased in peripheral blood and was associated with the severity of skin and lung fibrosis [5]. Infiltrating T cells in SSc skin and lungs were polarized toward a type-2 phenotype and were prone to produce profibrotic cytokines, including IL-4, IL-6, and IL-13 [6, 7]. Macrophages are the major producers of IL-6 and TGF-β, both of which have profibrotic functions in various organs. These profibrotic cytokines and growth factors activate and stimulate the differentiation of fibroblasts into α-smooth muscle actin (α-SMA)-positive myofibroblasts, leading to excessive production of extracellular matrix (ECM) in affected organs [8, 9].

Chemokines play a central role in the infiltration of leukocytes with the corresponding chemokine receptors into tissues. CX3CL1, also known as fractalkine, consists of a soluble chemokine domain and transmembrane domain [10–12]. CX3CL1 is expressed on the surface of various cell types, including endothelial cells, epithelial cells, macrophages, and vascular smooth muscle cells. Membrane-bound CX3CL1 on vascular endothelial cells selectively attracted mainly monocytes/macrophages and other cells including natural killer (NK) cells, and T cells via its cell surface receptor CX3CR1 and promoted their extravasation [12, 13]. Additionally, the soluble form of CX3CL1 (sCX3CL1) showed chemotactic effects on CX3CR1-positive cells, contributing to tissue-specific inflammation and fibrosis [14]. We and others reported increased serum sCX3CL1 levels in patients suffering from severe SSc with diffuse skin sclerosis, interstitial lung disease, or digital ulcers [15, 16]. Furthermore, our previous studies demonstrated that CX3CR1 deficiency or anti-CX3CL1 monoclonal antibody (mAb) therapy suppressed the development of bleomycin- and growth factor-induced skin fibrosis [17, 18]. However, the therapeutic effects of CX3CL1-CX3CR1 blockade on SSc-like inflammation and/or fibrosis in other organs remain unclear. Therefore, additional research using different mouse models of the complex immunopathology in SSc will allow further extrapolation of experimental findings to human clinical trials for SSc.

Chronic graft-versus-host disease (cGVHD) arises from alloreactive reactions between donor-derived immune and host cells. Transplantation of the bone marrow (BM) and splenocytes of B10.D2 mice (major histocompatibility complex haplotype: H-2^d^) into sublethally irradiated BALB/c mice (H-2^d^) across minor histocompatibility loci caused inflammation and subsequent fibrosis of the skin and lungs by 3 weeks after this procedure [19–21]. Therefore, the sclerodermatous cGVHD (Scl-cGVHD) mouse model has been widely used for assessing SSc pathogenesis and preclinical studies of SSc in addition to human Scl-cGVHD [22]. Anti-mouse CX3CL1 monoclonal antibody (anti-mCX3CL1 mAb) therapy selectively blocked intestinal infiltration of effector donor CD8^+^ T cells and attenuated acute graft-versus-host (GVH) reaction-associated intestinal injury without impairing graft-versus-tumor effects in mice [23]. However, the therapeutic effects of this treatment on the skin phenotype of Scl-cGVHD remain unclear. Therefore, this study evaluated the therapeutic utility of anti-CX3CL1 mAb in the Scl-cGVHD mouse model. Our findings indicated that the mAb exhibited anti-inflammatory and anti-fibrotic properties in both skin and lungs by inhibiting the recruitment of CX3CR1-expressing leukocytes and the subsequent production of proinflammatory and/or profibrotic molecules in lesional tissues.

## Methods

### Scl-cGVHD model

**Fig. 1:**
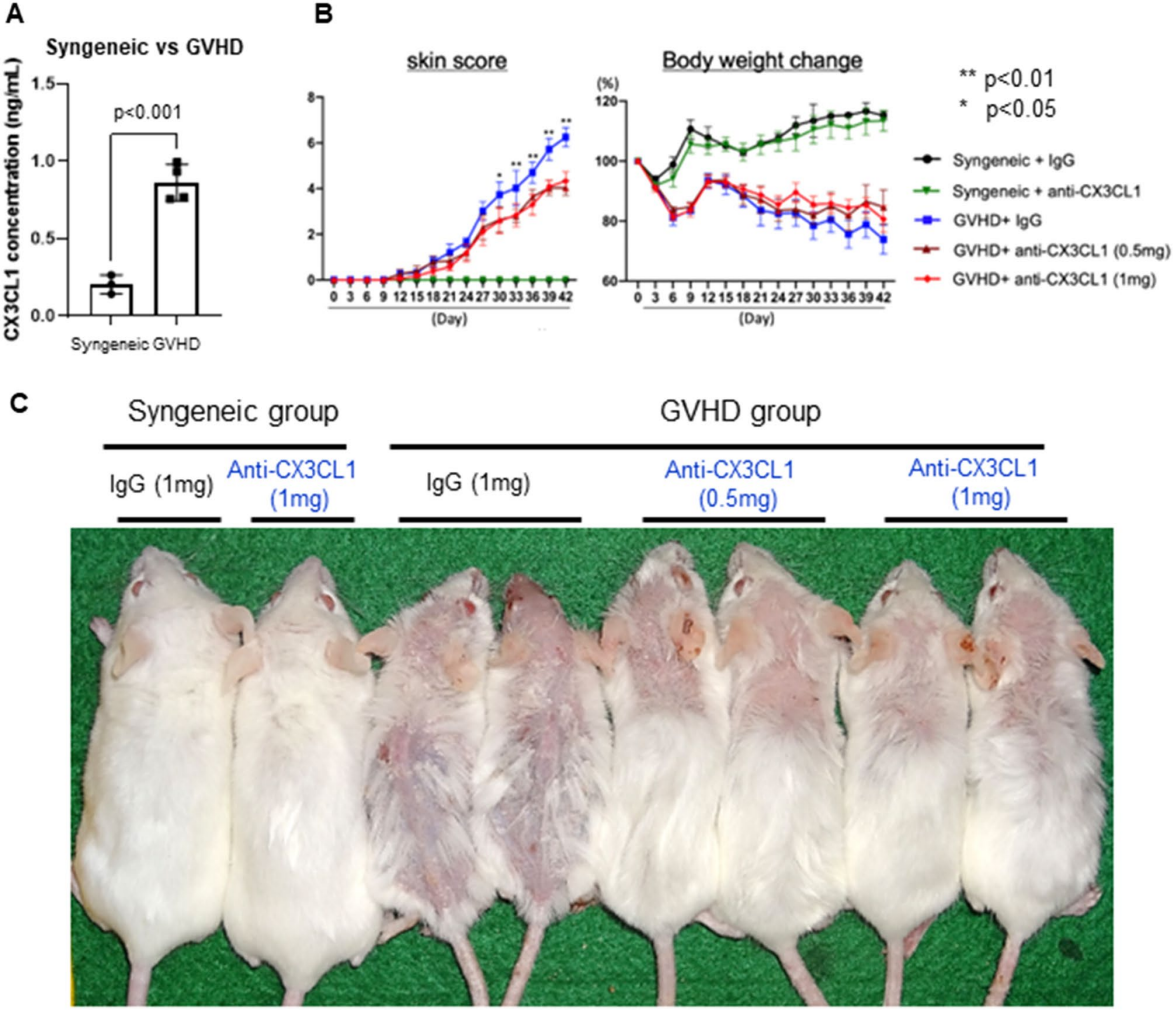
Anti-mouse CX3CL1 monoclonal antibody treatment attenuated skin severity in sclerodermatous chronic graft-versus-host disease model. **A** Serum levels of soluble CX3CL1 were measured by specific enzyme-linked immunosorbent assay (ELISA) in syngeneic group and sclerodermatous chronic graft-versus-host disease (GVHD) group. **B** Anti-mouse CX3CL1 monoclonal antibody (mAb) (0.5 mg or 1 mg) or control IgG was intraperitoneally injected into recipients from day 7 to day 35. **C** Skin scores and body weight change were monitored every 3 days. Values represented the mean ± standard error of the mean (SEM) of 3–10 mice per group. Photographs were taken 42 days after bone marrow transplantation

B10.D2 (H-2^d^) and BALB/c (H-2^d^) mice were purchased from CLEA (Japan). Mice were housed in a specific pathogen-free barrier facility. All experiments and procedures were approved by the Committee on Animal Experimentation of the University of Fukui (No. R01047). Eight- to 12-week-old male B10.D2 mice and female BALB/c mice were used as donors and recipients, respectively. BM was almost completely T cell-depleted (TCD) with anti-Thy1.2 microbeads (Miltenyi Biotech, Auburn, CA, USA) (Supple Fig. 1). BALB/c recipients were irradiated with 800 cGy (HW-200R; HITEX, Osaka, Japan) and injected via the tail vein with 10 × 10^6^ TCD-BM and 10 × 10^6^ splenocytes in 0.5 mL of phosphate buffered saline (PBS) to generate Scl-cGVHD (allogeneic bone marrow transplantation [BMT]). The control syngeneic group of female BALB/c mice received male BALB/c TCD-BM and splenocytes (syngeneic BMT) [21].

Allogenic and syngeneic recipients received an intraperitoneal injection of neutralizing anti-mCX3CL1 mAb (clone 5H8-4) [18, 24, 25] or control IgG (anti-DNP mAb) [26] at a dose of 0.5 mg or 1 mg twice a week from day 7 to day 35 after BMT.

### Skin score of cGVHD

Mice were weighed every 3 days after BMT and scored to assess the clinical severity of cGVHD skin as previously described [27]: healthy appearance, 0; skin lesions with alopecic area ≤1 cm^2^, 1; skin lesions with alopecic area of 1–2 cm^2^, 2; skin lesions with alopecic area of 2–5 cm^2^, 3; skin lesions with alopecic area of 5–10 cm^2^, 4; skin lesions with alopecic area of 10–15 cm^2^, 5; skin lesions with alopecic area of 15–20 cm^2^, 6; and skin lesions with alopecic area > 20 cm^2^, 7. Additionally, animals were assigned 0.4 points for skin disease (lesions or scaling) on the tail, and 0.3 points each for lesions on the ears and paws. The minimum and maximum scores were 0 and 8, respectively. Final scores for dead animals were kept in the dataset for the remaining time points of the experiment [21].

### Quantification of tissue fibrosis

Mouse skin and lungs were fixed in 10% formalin and embedded in paraffin. Serial Sect. (6-µm thickness) were stained with hematoxylin and eosin (H&E) and Masson’s trichrome. Dermal collagen thickness was measured from the epidermal-dermal junction to the lowermost dermis using light microscopy [21]. Masson’s trichrome blue-stained areas were pixelized and quantified using the ImageJ software (ver. 1.53, https://imagej.net/ij/). Total soluble collagen was quantified using the Sircol Soluble Collagen Assay (Biocolor, Belfast, UK) as previously described [22].

### Immunohistochemical staining of mouse skin and lungs

Section (6-µm thickness) from paraffin-embedded mouse skin and lungs were deparaffinized and incubated overnight at 4 °C with mouse mAbs to CD3 (1:200; Nichirei Bioscience), F4/80 (1:1600; Abcam), and α-SMA (1:200; DAKO), followed by incubation with peroxidase-labeled secondary antibody (Nichirei BioScience) and color development with the aminoethyl carbazole system (Nichirei BioScience) [28]. Immunostained cells were counted in three high-power microscopic fields. Each section was examined independently by two investigators (T.C. and N.O.) in a blinded manner.

### Immunofluorescence staining of mouse skin and lungs

Frozen skin Sect. (10-µm) were fixed for 10 min in 4% paraformaldehyde and treated with Blocking One Histo (Nacalai tesque, Cat:06349-64) for 1 h at room temperature, followed by overnight incubation at 4 °C with mouse mAbs against CX3CR1 (1:250) [26], F4/80 (1:500; Abcam:ab6640), or CD3 (1:500; Abcam: ab11089). Sections were then incubated with species-specific secondary antibodies conjugated with Alexa Fluor®488 (1:500; Invitrogen:R37118) or Alexa Fluor®568 (1:500; Life Technologies: A11007) for 30 min at room temperature and mounted in DAPI-containing Vectashield mounting medium (Vector:H-1800). Slides were visualized using a confocal laser scanning microscope (Olympus FV1200).

Immunoreactive cell numbers were counted visually in three randomly selected microscope fields in the dermis of a representative skin section from 3 to 6 mice in each group. The cell counting was performed blindly by two investigators (M.H. and N.O.).

### Enzyme-linked immunosorbent assay (ELISA)

Microtiter plates (NUNC, Porysorp) were coated with anti-mCX3CL1 mAb (clone 1H12) (KAN Research Institute, Inc., 5 µg/mL with 50 mM Tris/HCl [pH 7.5]) at 4°C overnight. After washing three times with the washing solution (50 mM Tris/HCl [pH 7.5], 150 mM NaCl, 0.01% Tween 20), wells were incubated with the blocking solution (50 mM Tris/HCl [pH 7.5], 150 mM NaCl, 0.01% Tween 20, 5% skim milk [Wako]) at 4°C overnight and then washed with the washing solution before use [18]. Samples or standard (recombinant mouse CX3CL1/Fractalkine, R&D) were added to the wells and incubated for 2 hours at room temperature. After washing three times with the washing solution, HRP-conjugated anti-mCX3CL1 mAb (clone #81) was added and incubated for 1 hour at room temperature. After efficient washing, the wells were incubated with a 3,3’5,5’-tetramethylbenzidine (TMB) Liquid Substrate System for ELISA (SIGMA) at 100 µL/well for 30 min at room temperature. To stop the reactions, 0.5 M H_2_SO_4_ was added at 100 µL/well, followed by color measurement at the optical density (OD) of 450–650 nm using an automated plate reader (ThermoMax, Molecular Device) [18].

### RNA sequencing and analysis

The mRNA sequencing (mRNA-Seq) library was prepared using the TruSeq Stranded mRNA LT Sample Prep Kit (Illumina) in accordance with the manufacturer’s instructions and optimized for Illumina Multiplexed Sequencing (https://targetepigenomics.org/docs/library_construction_protocol_perlab/Biswal_RNAseq_TruSeq.pdf). After purification of the amplified libraries, DNA quality of the products was assessed using the 2100 Bioanalyzer DNA 1000 assay. Paired-end sequencing of the mRNA-Seq libraries was performed using an Illumina NovaSeq6000 system (Illumina; 2 × 100 base paired-end runs) [28].

All raw sequencing reads were trimmed using the Trimmomatic software. Bases and quality control (QC) assessments of the sequencing were performed using FastQC. QC-passed reads were aligned to the Ensembl GRCm38.88 reference genome using Star v2.5.2b. The abundance of transcripts was then estimated using an expectation-maximization algorithm implemented in the software package RSEM v1.2.31 (https://github.com/deweylab/RSEM/) [29].

After primary analysis, differentially expressed gene (DEG) analysis was performed by using R with edgeR package. For statistical analysis, the Fisher’s exact test was used. DEGs were extracted using false discovery rate < 0.05 as cut off. The pathway analysis was performed by the Ingenuity Pathway Analysis software (IPA, QIAGEN). The statistical test was performed using Fisher’s exact test to assess the association between DEG sets and the canonical pathways. The significance of the association was evaluated by *p-values*.

### Statistical analysis

All data were presented as the mean ± standard error of the mean (SEM) and were analyzed using GraphPad Prism software version 7. Differences between samples were determined using the Student’s two-tailed t-test. P-values ≤0.05 were considered statistically significant.

## Results

### Serum sCX3CL1 levels were increased by Scl-GVHD induction

Previous reports showed increased serum sCX3CL1 levels in SSc patients with higher clinical severity and bleomycin-induced SSc mouse model [15, 16, 18]. First, we examined whether serum sCX3CL1 levels were affected by the development of the Scl-cGVHD phenotype in mice. On day 42 after BMT, serum sCX3CL1 levels showed a 4.2-fold increase in the Scl-cGVHD group as compared to the syngeneic control group (*p* < 0.001; Fig. 1A). Although the changes over time have not been confirmed, this result suggests the pathogenic involvement of CX3CL1 in this mouse model.

### Anti-CX3CL1 mAb treatment protected against dermal fibrosis in Scl-cGVHD model

To investigate the therapeutic utility of anti-CX3CL1 mAb in skin fibrosis in vivo, anti-CX3CL1 mAb was intraperitoneally injected into allogeneic BMT recipients twice a week from day 7 to day 35 after BMT. The degree of hair loss was known to correlate with the severity of skin fibrosis in the same region [27]. Skin fibrosis and alopecia appeared on day 18 after BMT in both control IgG-treated and anti-CX3CL1 mAb-treated groups and gradually became apparent. Both 0.5 mg and 1 mg doses of anti-CX3CL1 mAb treatment significantly reduced the increased rate of the Scl-cGVHD skin score as compared with control IgG administration (*p* < 0.01 at day 42; Fig. 1B, *left*). Both doses of anti-CX3CL1 mAb also slightly tended to inhibit cGVHD-dependent body weight loss, although the difference was not statistically significant (Fig. 1B, *right*). Photographic assessment demonstrated that control IgG-treated Scl-cGVHD mice showed mild and diffuse alopecia throughout the entire body surface on day 42 after BMT; however, the area and severity of alopecia were clearly reduced by both 0.5 mg and 1 mg doses of anti-CX3CL1 mAb treatment (Fig. 1C). No adverse events were evident in any of the anti-CX3CL1 mAb-treated syngeneic or cGVHD mice.

**Fig. 2:**
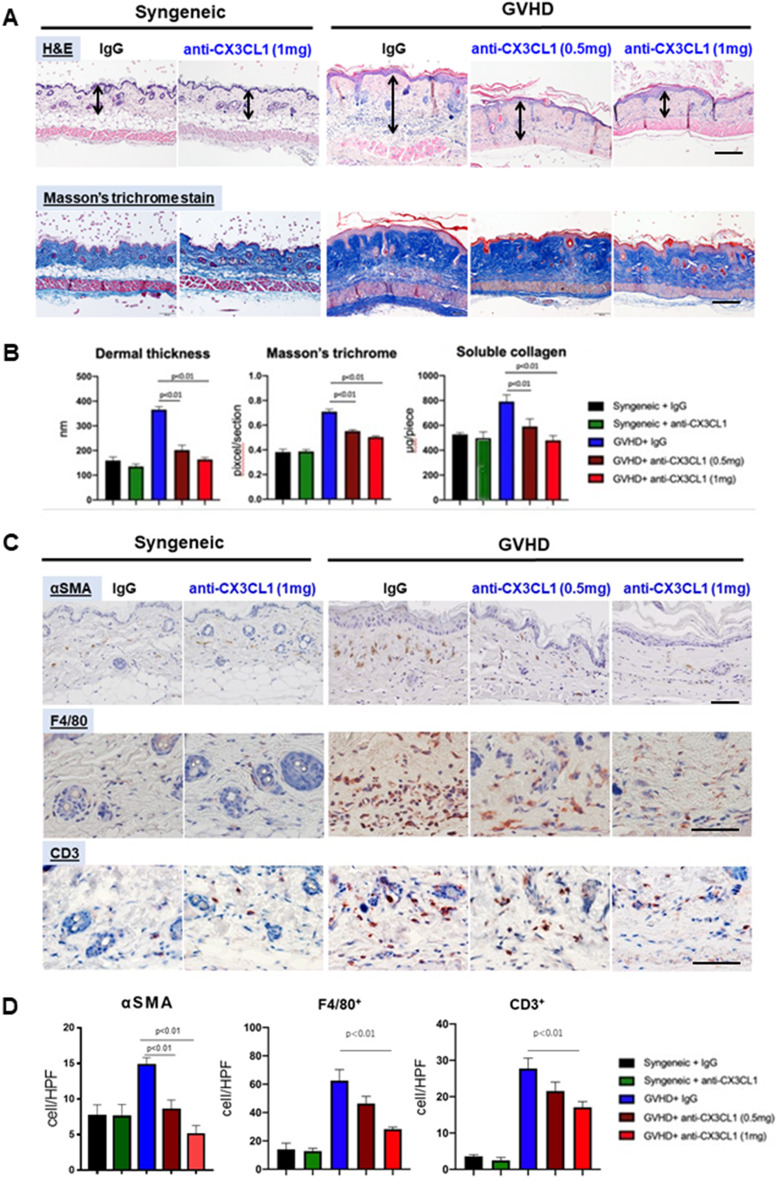
Anti-mouse CX3CL1 monoclonal antibody suppressed skin fibrosis and inflammation in sclerodermatous chronic graft-versus-host disease model. Skin tissues were harvested 42 days after bone marrow transplantation. **A** Representative histopathological changes of the skin due to anti-mouse CX3CL1 monoclonal antibody (mAb) or control IgG administration in sclerodermatous chronic graft-versus-host disease (Scl-cGVHD) mouse model. Arrows indicated dermal thickness. **B** Skin fibrosis was compared by determining dermal thickness, ratio of Masson’s trichrome-stained area to the total skin area, and soluble collagen content. **C, D** Paraffin sections were immunostained for α-SMA, F4/80, and CD3 (**C**) and quantified (**D**). Scale bar = 100 μm. Values represented mean ± standard error of the mean (SEM) of 3–5 mice per group

These findings were confirmed by histopathological examination using skin samples 42 days after BMT. Dermal thickening induced by cGVHD was significantly reduced in the both 0.5 mg and 1 mg doses of CX3CL1 mAb-treated group as compared to that in the control IgG group (*p* < 0.01, Fig. 2A, *upper*; B, *left*). Furthermore, altered dermal thickening was substantiated by a decrease in Masson’s trichrome-stained dermal fibrosis area in the CX3CL1 mAb-treated group as compared to that in the control IgG group (*p* < 0.01, Fig. 2A, *lower*; B, *middle*). Soluble collagen content in fibrotic skin was significantly decreased in the both 0.5 mg and 1 mg doses of CX3CL1 mAb-treated group as compared to that in the control IgG-treated group (*p* < 0.01, Fig. 2B, *right*). The overall therapeutic effects of anti-CX3CL1 mAb appeared to show a dose-dependent trend. Therefore, anti-CX3CL1 mAb treatment significantly reduced the skin fibrosis of Scl-cGVHD mice.

### Anti-CX3CL1 mAb treatment decreased myofibroblasts, macrophages, and T cells in the skin of Scl-cGVHD model

To evaluate fibrogenic and inflammatory infiltrating cells in the skin of Scl-cGVHD mice, we performed immunostaining with a panel of antibodies using skin samples 42 days after BMT. The number of α-SMA-positive myofibroblasts, which play a central role in collagen synthesis, was markedly increased in fibrotic skin. Anti-CX3CL1 mAb treatment significantly reduced this increase in a dose-dependent manner (1 mg: *p* < 0.01, 0.5 mg: *p* < 0.05, Fig. 2C, *upper*; D, *left*). The numbers of F4/80^+^ macrophages and CD3^+^ T cells were reduced in CX3CL1 mAb-treated skin as compared to those in control IgG-treated skin. In particular, the administration of a high-dose mAb (1 mg/body) significantly inhibited the infiltration of these cells (*p* < 0.01, Fig. 2C, *middle and lower*; D, *middle and right*). Thus, anti-CX3CL1 mAb therapy reduced the fibrogenic cell and immune cell numbers in Scl-cGVHD mouse skin.

### Anti-CX3CL1 mAb therapy was effective for lung fibrosis in Scl-cGVHD model

**Fig. 3:**
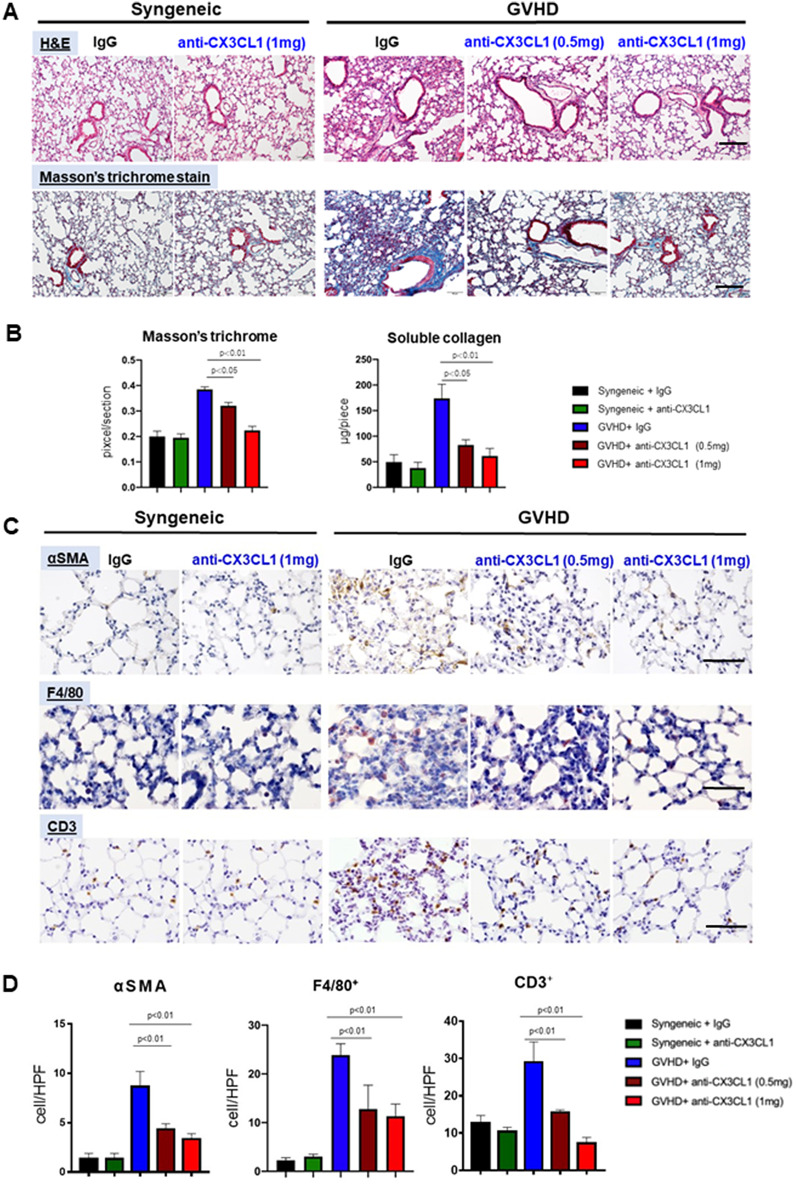
Anti-mouse CX3CL1 monoclonal antibody inhibited lung fibrosis and inflammation in sclerodermatous chronic graft-versus-host disease model. Lung tissues were harvested 42 days after bone marrow transplantation. **A** Representative histopathological changes in the lungs due to anti-mouse CX3CL1 monoclonal antibody (mAb) or control IgG administration in sclerodermatous chronic graft-versus-host disease (Scl-cGVHD) mouse model. **B** Lung fibrosis was compared by determining the ratio of trichrome-stained area to the total area and soluble collagen content. **C, D** Paraffin sections were immunostained for α-SMA, F4/80, and CD3 (**C**) and quantified (**D**). Scale bar = 100 μm. Values represented mean ± standard error of the mean (SEM) of 3–5 mice per group

We next evaluated the efficacy of CX3CL1 antagonism for lung fibrosis induced by cGVHD using lung samples 42 days after BMT. As observed in the Scl-cGVHD skin, Masson’s trichrome-stained fibrotic areas in the lung interstitium were significantly reduced by anti-CX3CL1 mAb treatment as compared to control IgG treatment. This effect was more prominent with a high-dose mAb (1 mg/body) than with a low-dose mAb (*p* < 0.05 and *p* < 0.01, respectively; Fig. 3A and B, *left*). These results were consistent with the collagen assay using whole lung homogenates, which showed a marked decrease in soluble collagen content following anti-CX3CL1 mAb treatment (1 mg: *p* < 0.01, 0.5 mg: *p* < 0.05; Fig. 3B, *right*) as compared to control IgG injection. Therefore, anti-CX3CL1 mAb treatment significantly inhibited the lung fibrosis of Scl-cGVHD mice.

### Anti-CX3CL1 mAb treatment reduced myofibroblasts, macrophages, and T cells in the lungs of Scl-cGVHD model

Immunohistochemistry using Scl-cGVHD lungs (day 42) showed that the increased numbers of α-SMA-positive myofibroblasts, F4/80^+^ macrophages, and CD3^+^ T cells were significantly attenuated by anti-CX3CL1 mAb treatment (*p* < 0.01, Fig. 3C, D). The responses tended to be dose-dependent, particularly with a decrease in the number of CD3^+^ T cells that approached the control level in syngeneic mice. Thus, anti-CX3CL1 mAb treatment significantly reduced the fibrogenic cell and immune cell numbers in the lungs of Scl-cGVHD mice, as was a similar observation in the skin.

### CX3CR1-positive macrophages and T cells infiltrating the skin and lungs of Scl-cGVHD mice were decreased by anti-CX3CL1 mAb treatment

**Fig. 4:**
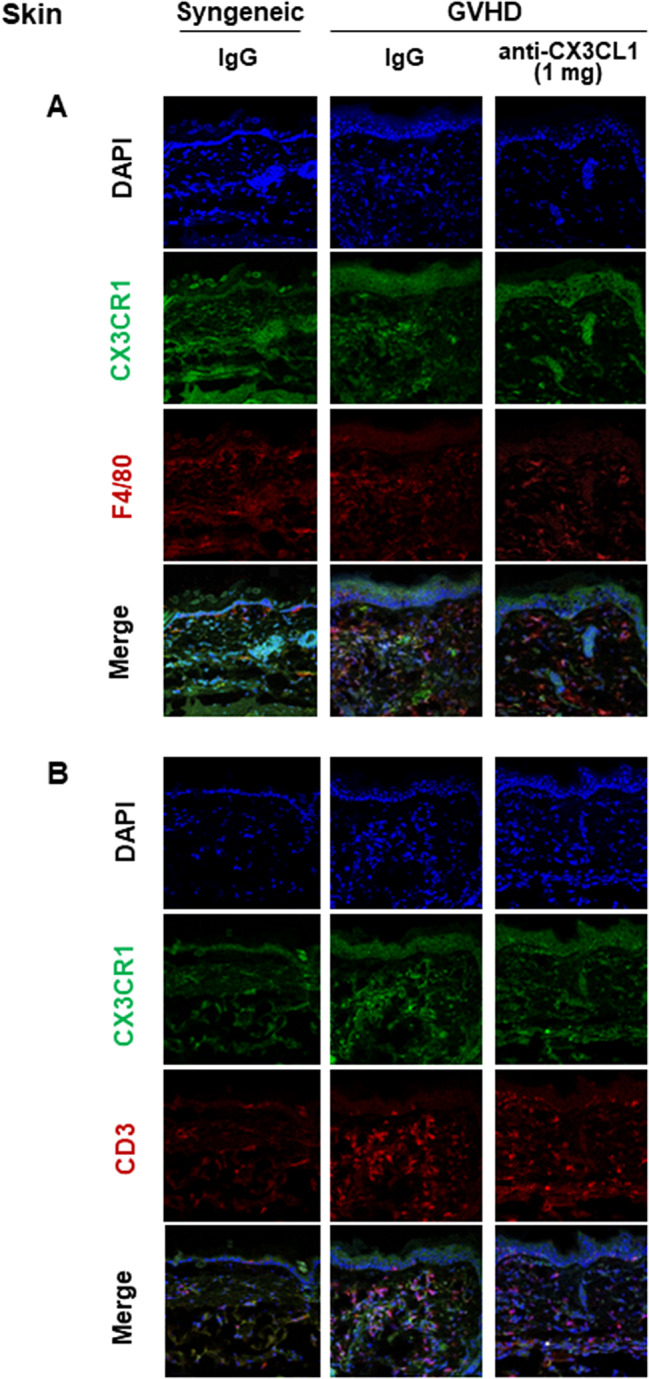
Anti-mouse CX3CL1 monoclonal antibody reduced skin infiltration of CX3CR1-expressing leukocytes in sclerodermatous chronic graft-versus-host disease. Representative images of CX3CR1 and F4/80 or CD3 staining of frozen skin tissues 14 days after bone marrow transplantation in 3 groups (syngeneic group treated with control IgG, sclerodermatous chronic graft-versus-host disease (Scl-cGVHD) group treated with IgG, and Scl-cGVHD group treated with anti-CX3CL1 mAb). *n* = 4–6 mice per group

We used fluorescent immunostaining to determine whether macrophages and T cells increased in the skin and lungs of Scl-cGVHD express CX3CR1, the receptor for CX3CL1, and whether anti-CX3CL1mAb inhibits their cell infiltration in the process of fibrosis (day 14 after BMT). Cells positive for both CX3CR1 and F4/80 were increased in the skin with Scl-cGVHD, but were significantly suppressed by 1 mg mAb treatment (*p* < 0.01, Fig. 4 A). CX3CR1 and CD3 double-positive cells were also increased in Scl-cGVHD-affected skin, but were significantly inhibited by 1 mg of mAb therapy (*p* < 0.01, Figs. 4B and 6A). The percentage of CX3CR1 expression in F4/80 or CD3 positive cells was increased by cGVHD but significantly suppressed by mAb treatment (*p* < 0.05, respectively, Figs. 4 and 6A).

**Fig. 5:**
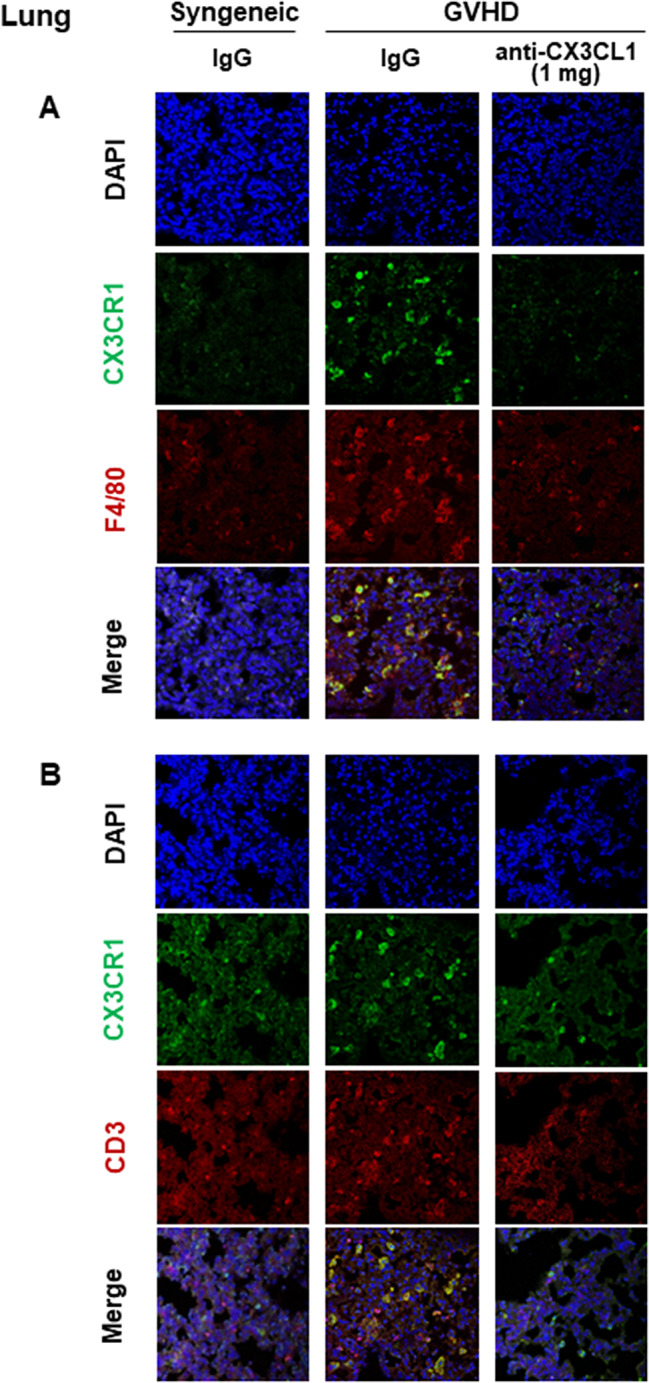
Anti-mouse CX3CL1 monoclonal antibody reduced lung infiltration of CX3CR1-expressing leukocytes in sclerodermatous chronic graft-versus-host disease. Representative images of CX3CR1 and F4/80 or CD3 staining of frozen lung tissues 14 days after bone marrow transplantation in 3 groups (syngeneic group treated with control IgG, Scl-cGVHD group treated with IgG, and Scl-cGVHD group treated with anti-CX3CL1 mAb)

**Fig. 6:**
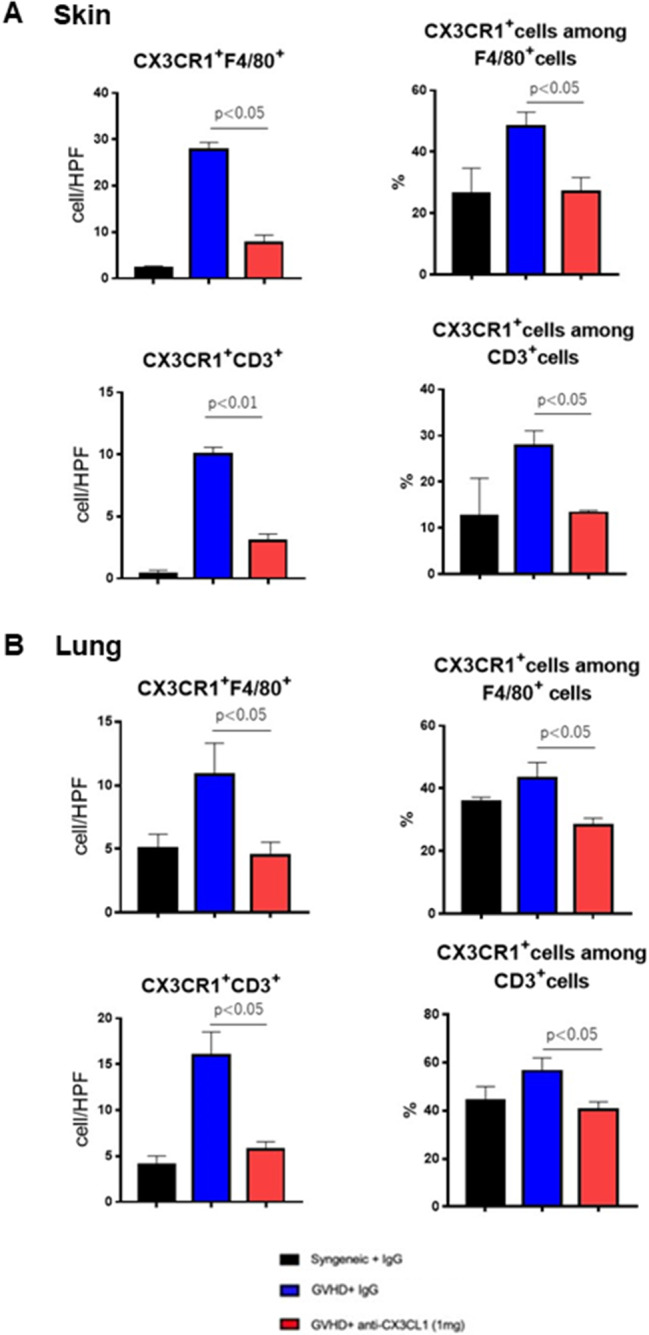
Anti-mouse CX3CL1 monoclonal antibody therapy suppressed infiltration of leukocytes, preferentially CX3CR1-expressing leukocytes, in the skin and lungs of sclerodermatous chronic graft-versus-host disease. Analysis of CX3CR1 and F4/80 or CD3 staining in the frozen skin (**A**) and lung tissues (**B**) from sclerodermatous chronic graft-versus-host disease mice 14 days after bone marrow transplantation. The numbers of CX3CR1 and F4/80 double-positive cells and CX3CR1 and CD3 double-positive cells were counted visually in 3 randomly selected microscope high-power field (x400) in the skin and lung section in each group using Photoshop software (Adobe). The proportion of CX3CR1-expressing cells among F4/80 or CD3-positive cells was also quantified. Values represented the mean ± standard error of the mean (SEM) of 4–6 mice per group

Similarly, in the lungs, CX3CR1 and F4/80 double-positive cells and CX3CR1 and CD3 double-positive cells were increased in Scl-cGVHD, but were significantly suppressed by both 1 mg of CX3CL1 mAb (*p* < 0.05, Figs. 5 and 6B). The proportion of CX3CR1 expression in F4/80- or CD3-positive cells were significantly decreased by 1 mg of mAb therapy (*p* < 0.05, Figs. 5 and 6B). Thus, anti-CX3CL1 mAb preferentially inhibited CX3CR1-positive leukocyte infiltration in the skin and lungs of Scl-cGVHD mice.

### Anti-CX3CL1 mAb administration inhibited the upregulation of proinflammatory and profibrotic gene sets in the skin of Scl-cGVHD mice

**Fig. 7:**
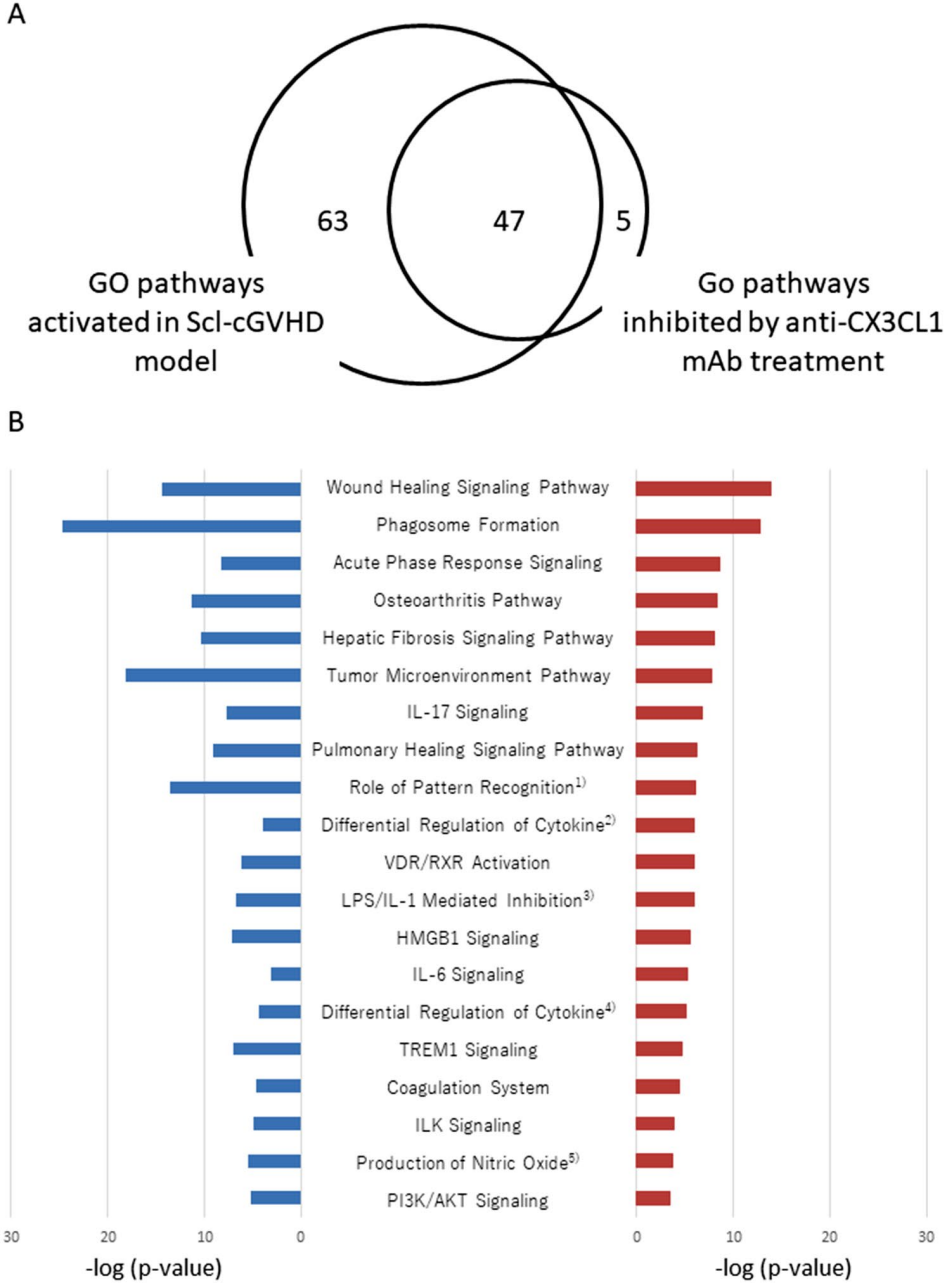
Pathway analysis of skin gene expression in sclerodermatous chronic graft-versus-host disease. Pathway analysis of RNA sequencing data was performed to determine changes in molecular functions that were responsible for antibody treatment-induced improvement. **A** The Venn diagram shows the number of canonical gene ontology (GO) pathways that were activated in sclerodermatous chronic graft-versus-host disease (Scl-cGVHD) model and/or were inhibited by anti-FKN mAb treatment. **B** Among the 47 GO pathways that were activated in Scl-cGVHD and were also inhibited by mAb treatment, the top 20 pathways with the highest significance in the inhibition after mAb treatment were shown. Blue bars indicated the *p*-value, from which the significance of the association magnitude of between the increase in gene expression in Scl-cGVHD skin as compared to control syngeneic skin and each canonical pathway was determined. Red bars showed the *p*-value, from which the significance between magnitude of the decrease in gene expression after antibody treatment and each canonical pathway was determined. (1) Role of Pattern Recognition Receptors in Recognition of Bacteria and Viruses, (2) Differential Regulation of Cytokine Production in Macrophages and T Helper Cells by IL-17 A and IL-17 F, (3) LPS/IL-1 Mediated Inhibition of RXR Function, (4) Differential Regulation of Cytokine Production in Intestinal Epithelial Cells by IL-17 A and IL-17 F, (5) Production of Nitric Oxide and Reactive Oxygen Species in Macrophages

Finally, we attempted to analyze the gene expression profile in Scl-cGVHD fibrotic skin lesions (day 42) using RNA sequencing to evaluate the types of molecular function changes, which were responsible for the improvement in phenotypic outcomes with anti-CX3CL1 mAb treatment. Ingenuity Pathway Analysis (IPA) efficiently identified the canonical gene ontology (GO) pathways that were activated in Scl-cGVHD and/or were inhibited by mAb treatment (Fig. 7A): the former included 120 functional gene categories and the latter were 52 ones. Among the 47 common GO pathways that were activated in Scl-cGVHD and were also inhibited by mAb treatment, their top 20 pathways with the highest significance in the inhibition after mAb treatment were shown (Fig. 7B). Gene profiles associated with tissue fibrosis and/or remodeling and inflammation were variable in consistent with phenotypic changes following anti-CX3CL1 mAb treatment of the skin of Scl-cGVHD mice. Pathways related to tissue fibrosis included the wound healing signaling pathway, phagosome formation, hepatic fibrosis signaling pathway, IL-17 signaling pathway, pulmonary healing signaling pathway, differential regulation of cytokine production in macrophages, VDR/RXR activation, LPS/IL-1 mediated inhibition of RXR function, HMGB1 signaling, and IL-6 signaling pathway (Fig. 7B, *red bars*). Additionally, inflammatory signaling pathways, such as phagosome formation, acute phase response signaling, tumor microenvironment, IL-17 signaling, and differential regulation of cytokine production in macrophages, were also listed in the top 20. Therefore, anti-CX3CL1 mAb effectively inhibited the upregulation of proinflammatory and profibrotic genes in the skin of Scl-cGVHD mice.

## Discussion

The current study demonstrated that neutralizing anti-CX3CL1 mAb inhibited the progression of both skin and lung fibrosis in the Scl-cGVHD mouse model. No apparent adverse effects were observed in any treatment groups. In skin and lung tissues, the predominant infiltration of CX3CR1-expressing macrophages and CX3CR1-expressing T cells was significantly reduced by anti-CX3CL1 mAb treatment. Gene expression profiles altered by anti-CX3CL1 mAb therapy exhibited the upregulation of proinflammatory and profibrotic genes in the lesional skin of Scl-cGVHD mice.

The Scl-cGVHD mouse model showed early infiltration of macrophages and/or T cells, and subsequent tissue fibrosis in both skin and lungs. These symptoms are to some extent similar to SSc in humans, but in this mouse model there is alopecia in the fibrotic areas of the skin. In this mouse model, sCX3CL1 levels were markedly elevated after Scl-cGVHD induction. Therefore, we evaluated the effect of anti-CX3CL1 mAb therapy on Scl-cGVHD. The mAb treatment significantly suppressed skin and lung fibrosis in Scl-cGVHD model. Most infiltrated leukocytes in the skin and lungs of Scl-cGVHD were CX3CR1 and F4/80 double-positive cells and CX3CR1 and CD3 double-positive cells. MAb treatment significantly reduced the number of F4/80-positive and CD3-positive cells in the skin and lungs and the percentage of CX3CR1 expression in these cells.

Among gene sets upregulated by Scl-cGVHD, RNA sequencing demonstrated that most pathways of genes downregulated by mAb treatment were associated with tissue remodeling, macrophages, innate immunity, and the IL-6/IL-17 signals in the skin. Additionally, inflammatory signaling pathways, such as phagosome formation, acute phase response signaling, tumor microenvironment, IL-6/IL-17 signaling, and differential regulation of cytokine production in macrophages, were also listed in the top 20. These RNA sequencing data also support the pathogenic perturbation of locally infiltrating and activating macrophages in the tissue fibrosis of Scl-cGVHD. Of note, these macrophage-related gene clusters can be activated in part via innate immune responses, such as phagosome formation, IL-6 and IL-17 signaling, and pathogen-pattern recognition receptors, which were all downregulated after anti-CX3CL1 mAb treatment. Gene expression analysis demonstrated that both M1 and M2 macrophages were up-regulated in the skin of early diffuse cutaneous SSc patients [30]. Therefore, profibrotic monocytes/macrophages and molecules affecting the differentiation and/or activation of them are considered therapeutic targets of SSc [4, 31]. Previous findings indicate that CX3CL1-CX3CR1 interaction is critical for monocyte/macrophage homeostasis and differentiation and regulates the fate of monocyte/macrophage-derived cells in a variety of inflammatory diseases including fibrotic diseases [32, 33]. Thus, our findings suggest that anti-CX3CL1 mAb therapy that inhibits the infiltration of macrophages into the tissue is a rational strategy for the treatment of skin fibrotic disorders, such as Scl-cGVHD and/or SSc.

Previous studies demonstrated that CX3CL1-CX3CR1 interaction was important for the development of fibrosis in various organs, including lungs, liver, kidney, and peritoneum [34–37]. Ishida et al. indicated that locally produced CX3CL1 could exacerbate bleomycin-induced pulmonary fibrosis, primarily by recruiting CX3CR1-expressing M2 macrophages and fibrocytes into the lungs [34]. Helmke et al. reported that crosstalk between macrophages and mesothelial cells via CX3CR1-CX3CL1 interaction promoted mesothelial TGF-β production and subsequent peritoneal fibrosis in response to dialysate exposure in mice [38]. Shimizu et al. demonstrated that the CX3CL1-CX3CR1 axis contributed to hypertensive kidney fibrosis, possibly by enhancing macrophage infiltration and TGF-β1 expression in a mouse model [37]. In contrast, the dendritic cell-independent kidney fibrosis model of unilateral ureteral obstruction was exacerbated via local proliferation of profibrotic macrophages in CX3CR1-deficient mice [39]. Therefore, utilization of the blockade of CX3CL1-CX3CR1 axis should be carefully evaluated for individual fibrotic diseases.

In our previous study, simultaneous treatment with anti-mCX3CL1 mAb suppressed the development of skin fibrosis in both bleomycin- and growth factor-induced skin fibrosis [18]. Moreover, the progression of bleomycin- or growth factor-induced skin fibrosis was significantly protected in the skin of CX3CR1-deficient mice [17]. Additionally, findings of the current study indicated that this treatment might be useful for skin and lung fibrosis in SSc using another mouse model with a different mechanism of pathogenesis.

This study has several limitations. It seems necessary to confirm the prognostic effect of this antibody treatment on survival in this model of GVHD. We should also confirm the therapeutic efficacy and side effects when higher doses of the mAb are used. This study did not provide a complete picture of the mechanisms by which CXCL1-targeted therapy affects the disease state. More detailed and larger-scale analyses and further investigation including cells and specimens from SSc patients are needed to confirm the utility of anti-CX3CL1 mAb.

## Conclusions

In summary, our findings in a mouse model indicated that the CX3CL1-CX3CR1 pathway was important for the development of Scl-cGVHD. Humanized anti-CX3CL1 mAb might be a potential candidate for clinical trials of SSc and Scl-cGVHD.

### Electronic supplementary material

Below is the link to the electronic supplementary material.

Supplementary Material 1

## Data Availability

The datasets used and/or examined for the current study will be available from the corresponding author upon reasonable request. RNA sequence data are submitted to the Gene Expression Omnibus (GEO). The data is available under the accession number GSE227285.

## Abbreviations

α-SMA: α-smooth muscle actin
anti mCX3CL1 mAb: anti-mouse CX3CL1 monoclonal antibody
BM: bone marrow
BMT: bone marrow transplantation
cGVHD: chronic graft-versus-host disease
CX3CL1: fractalkine
ECM: extracellular matrix
ELISA: enzyme-linked immunosorbent assay
GO: gene ontology
GVH: graft-versus-host
GVHD: graft-versus-host disease
H&E: hematoxylin and eosin
IL-4RA: IL-4-receptor α
mAb: monoclonal antibody
mRNA-Seq: mRNA sequencing
NK: natural killer
OD: optical density
PBS: phosphate buffered saline
QC: quality control
rpm: rotations per minute
Scl-cGVHD: sclerodermatous chronic graft-versus-host disease
sCX3CL1: soluble form of CX3CL1
SEM: standard error of the mean
Spp1: osteopontin
SSc: systemic sclerosis
TCD: T cell-depleted
TGF: transforming growth factor
TMB: tetramethylbenzidine
