# Supplementary figures and images for: Anti-CX3CL1 (fractalkine) monoclonal antibody attenuates lung and skin fibrosis in sclerodermatous graft-versus-host disease mouse model
Source: Arthritis Res Ther. 2024 May 3;26:94. doi: 10.1186/s13075-024-03307-8 (PMC11067205; doi:10.1186/s13075-024-03307-8)

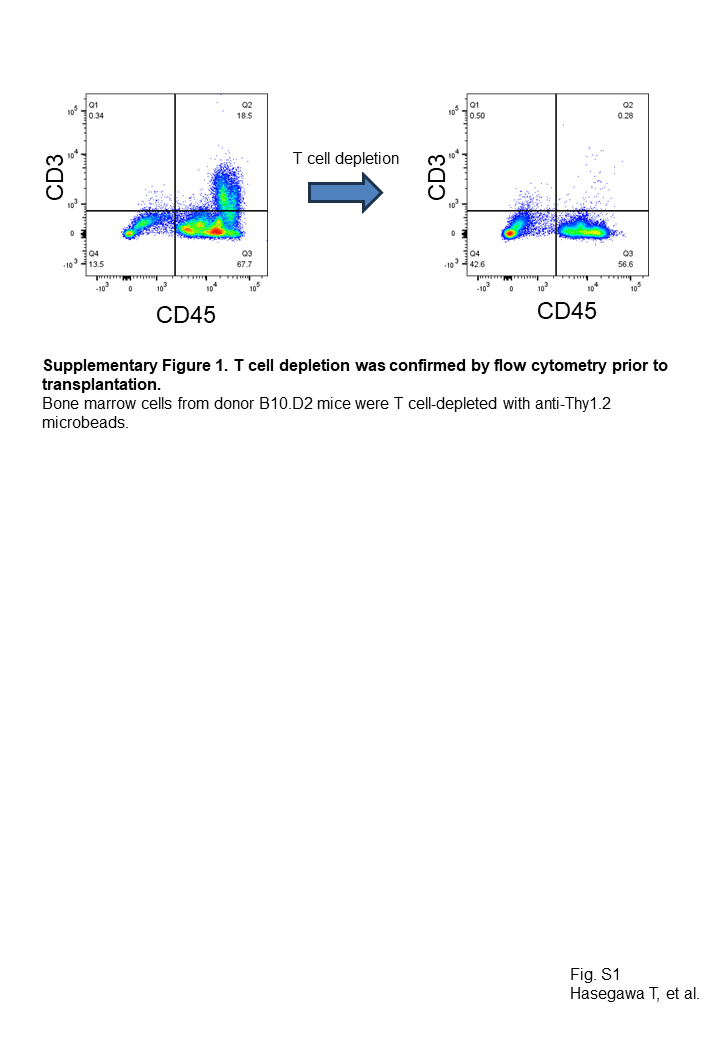

Supplement: Supplementary file 1 — Supplementary Material 1 [file 13075_2024_3307_MOESM1_ESM.tif]
